# Patent anomalous circumflex coronary artery stent occlusion following aortic valve replacement with coronary artery bypass

**DOI:** 10.21542/gcsp.2022.12

**Published:** 2022-06-30

**Authors:** Shrey Kapoor, Katherine Giuliano, Eric Etchill, Hamza Aziz, Jennifer S. Lawton

**Affiliations:** Division of Cardiac Surgery, Department of Surgery, Johns Hopkins University, Baltimore, Maryland, USA

## Abstract

An anomalous left circumflex artery branching arising from the right coronary artery is one of the most common congenital coronary artery abnormalities. Despite this, the incidence is low and our clinical understanding of the nuances in patients with such abnormalities remains limited. We present a case of a 73-year-old male with coronary artery disease status-post stenting of an anomalous circumflex artery who subsequently underwent coronary artery bypass grafting and surgical aortic valve replacement with EKG changes post-operatively. He was emergently taken to the cardiac catheterization lab, where catheterization revealed total occlusion of the proximal circumflex artery, just distal to the previous stent. Acute inferior ST-elevation myocardial infarction was suspected to be secondary to intraoperative external manipulation at the site of occlusion in the retro-aortic segment of the vessel. In patients with abnormal coronary artery anatomy, it is imperative to monitor for new EKG changes that may be indicative of new ischemia requiring further intervention.

## Case Presentation

The patient was a 73-year-old male with a past medical history of coronary artery disease with a prior inferolateral ST segment elevation myocardial infarction complicated by ventricular fibrillation arrest. In 2012, he underwent catheterization and percutaneous coronary intervention (PCI) with placement of a 3.5 mm × 18 mm everolimus-eluting stent to an anomalous left circumflex artery originating from the right coronary artery (RCA) and coursing in a retro-aortic position. Additional medical history included chronic atrial fibrillation (on apixaban), hypothyroidism, hyperlipidemia, hypertension, GI bleed due to arteriovenous malformation, obesity, and a 20 pack-year history of tobacco use.

**Figure 1.**
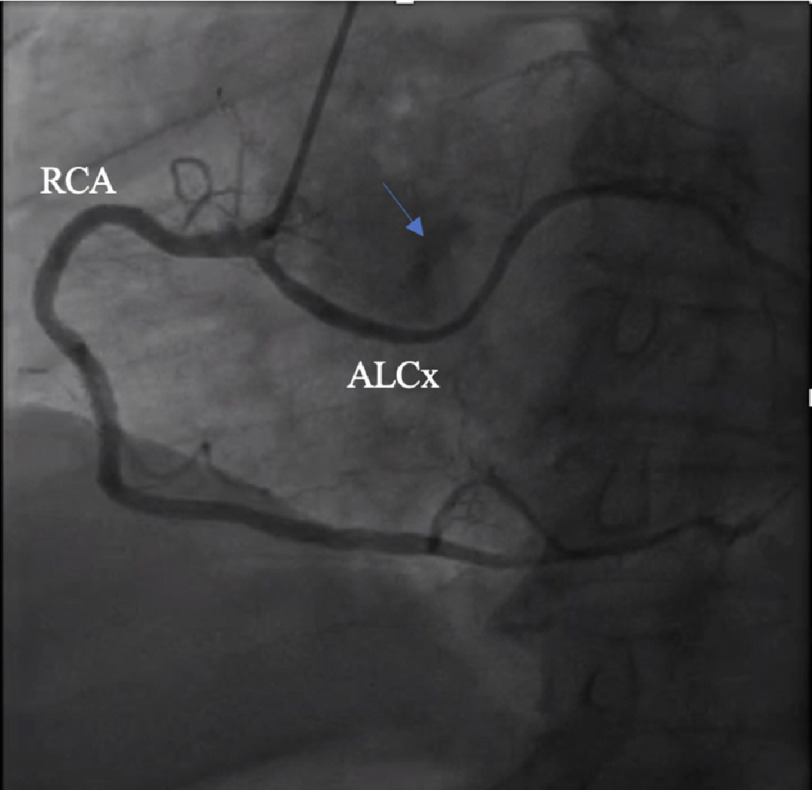
Pre-operative coronary catheterization revealed an anomalous left circumflex artery (ALCx) originating from the right coronary artery (RCA) with 30% proximal stenosis and a patent stent. The RCA showed <30% stenosis. Calcification of the aortic valve also demonstrated (*blue arrow*).

**Figure 2.**
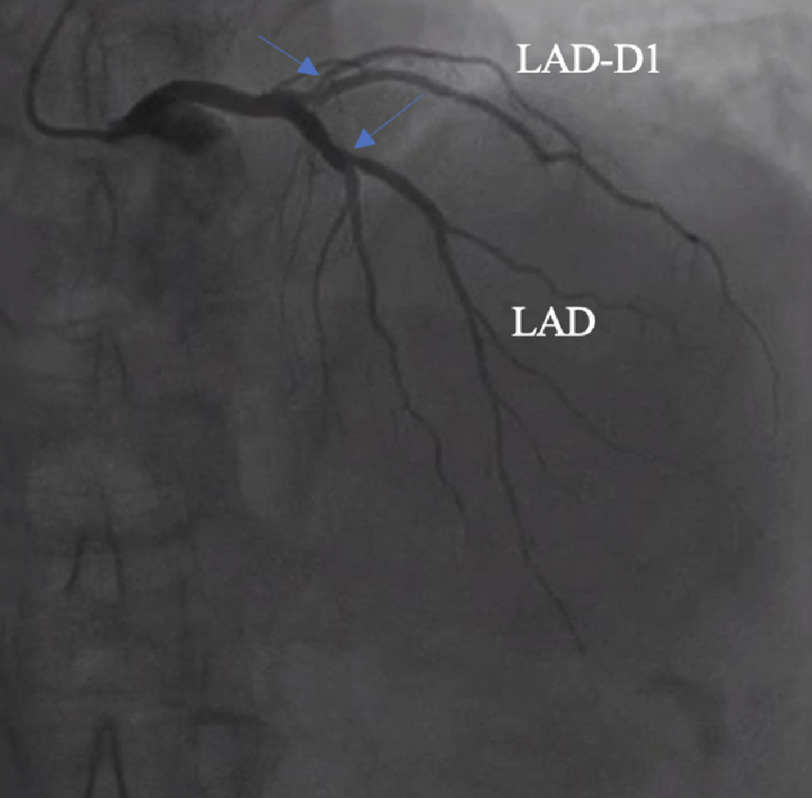
Pre-operative coronary catheterization demonstrating the left anterior descending (LAD) artery with 60% mid-stenosis (blue arrow), and diagnonal-1 with 60–70% ostial stenosis (blue arrow).

Six years after his initial PCI, he presented with new onset dyspnea on exertion. A transthoracic echocardiogram (TTE) demonstrated a bicuspid aortic valve with an aortic valve area of 0.6 cm^2^, mean gradient of 45 mmHg, peak gradient of 72 mmHg, and peak velocity of 4.3 m/s, consistent with severe aortic stenosis. TTE additionally demonstrated mild-to-moderate aortic and mitral regurgitation and normal left ventricular ejection fraction (LVEF) of 55–60%. He underwent coronary catheterization, which was significant for a dominant RCA with <30% stenosis, retro-aortic circumflex with 30% proximal stenosis and patent stent (Figure 1), left anterior descending (LAD) with 60% mid stenosis, and LAD diagonal-1 (diag1) with 70–80% ostial stenosis (Figure 2).

The patient underwent aortic valve replacement with a 25 mm 2800 series bovine pericardial aortic valve and coronary artery bypass grafting (CABG) x2 with the left internal mammary artery (LIMA) to the LAD and the saphenous vein graft (SVG) to the diagonal. Total cardiopulmonary bypass time was 184 min and the total crossclamp time was 160 min. A polyester net heart-positioning device was used. Spontaneous resumption of normal sinus rhythm was observed. Intraoperative EKG monitoring was notable for inferior lead ST-segment elevation, which was attributed to air in the left ventricle. The EKG changes improved, and the intraoperative transesophageal echocardiogram (TEE) demonstrated appropriate function of the aortic valve with no peri-valvular leak, an LVEF of 50–55%, and no wall motion abnormalities.

**Figure 3.**
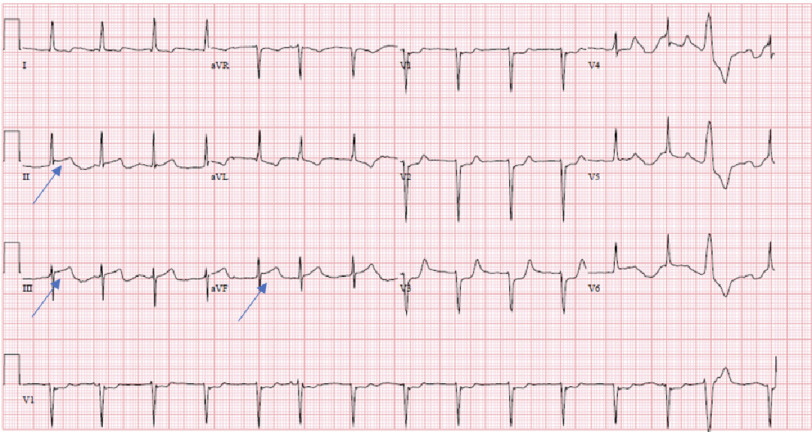
Post-operative EKG demonstrating ST-segment elevation in leads II, III, and aVF (*blue arrows*).

**Figure 4.**
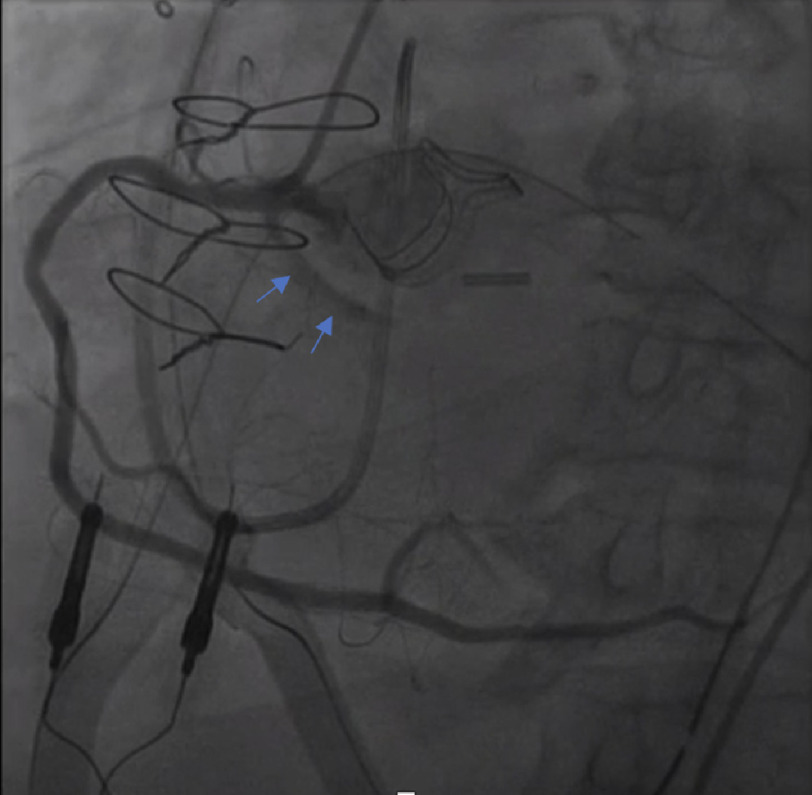
Post-operative cardiac catheterization demonstrating the right coronary artery with the anomalous circumflex artery; the previously placed stent was occluded (blue arrows) with no distal flow through the course of the circumflex artery. The aortic prosthesis is visible near the stent. Also visible are a Swan-Ganz catheter, pacing wires, chest tubes, and sternal wires.

**Figure 5.**
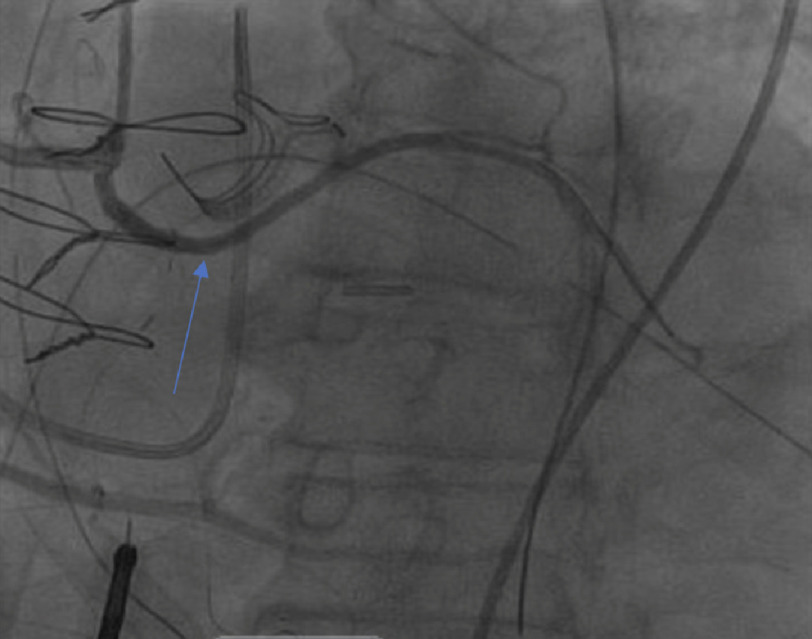
Post-operative cardiac catheterization demonstrating the new stent and close proximity of the newly opened vessel to the aortic valve prosthesis.

He was transferred to the intensive care unit (ICU), where - on post-operative day 0 - he was noted to have worsening acidosis and ST segment elevation in leads II, III, and aVF (Figure 3). He was emergently taken to the cardiac catheterization lab, where catheterization revealed total occlusion of the proximal circumflex artery, just distal to the previous stent (Figure 4). Both bypass grafts were widely patent. This was treated with PCI with placement of a drug-eluting 2.5  × 30 mm stent (Figure 5), with thrombolysis in myocardial infarction (TIMI) 3 flow, and without residual stenosis. Acute inferior STEMI (ST-elevation myocardial infarction (STEMI) was suspected secondary to intraoperative external compression/manipulation at the site of occlusion in the retro-aortic segment of the vessel. Post-PCI, he was started on ticagrelor. He self-extubated 10 days after the PCI. Three weeks after the operation, the patient experienced episodes of complete heart block, for which he received a permanent pacemaker. He was discharged for acute rehabilitation on post-operative day 32. On follow-up in clinic one-month post-discharge, he was doing well with some dyspnea on exertion but no chest pain or shortness of breath at rest. His post-op TTE showed normal left ventricular ejection fraction and a normal functioning aortic valve with no paravalvular leak.

## Literature Review

Congenital coronary artery abnormalities are present in 1% of the population, with the most common variation being an anomalous left circumflex artery (ALCx) occurring in up to 0.7% of patients^1^. This anomalous artery can either arise from a separate ostium in the right sinus or, even more unusually, as a branch off of the proximal right coronary artery (RCA). Some authors have suggested that patients with anomalous coronary arteries are at a greater risk of contortion of their arteries, thereby compounding their risk of ischemia and arrhythmias^2^.

The reported patient’s ALCx originated from the RCA, and when he presented with symptomatic aortic stenosis, he had already undergone PCI with stent placement in this ALCx. He subsequently underwent surgical aortic valve replacement and coronary artery bypass grafting x2. The patient experienced post-operative ST changes, and catheterization revealed total occlusion of the proximal circumflex artery.

The significance of post-operative ST segment changes is widely variable as the etiology of these changes can be non-specific. The differential diagnosis for such changes includes post-operative inflammatory changes, myocarditis, pericarditis, or acute ischemia. Post-pericardiotomy syndrome (PPS), which is triggered by an inflammatory response following damage to the pericardium, occurs after 10–40% of cardiac operations^3^. The incidence of post-operative MI is 2–15% of all cardiac surgery cases, which can be either graft or non-graft related, but the index of suspicion must be high in the post-operative patient with EKG changes^4,5^. A myocardial infarction post-CABG is classified as type 5^6^. Evaluation of these patients is made more challenging because echocardiographic evaluation can be poor due to its modest resolution, particularly in an early post-operative patient with a sternal wound, wires, and chest tubes. Furthermore, in this immediate post-operative period, many patients remain intubated and sedated, making it difficult or impossible to properly assess for symptoms. Potential treatment options include a return to the operating room or coronary catheterization for diagnosis and potential intervention.

Elevation of cardiac troponin values, the development of q waves, angiographically documented artery occlusion, or echocardiography for wall motion abnormalities are the current modalities for evaluating a post-operative MI^6^. Although ST segment elevations are not reliable indications of a post-operative MI, this case of an occluded anomalous left circumflex artery following CABG indicates that ST changes can still represent acute arterial occlusion. In addition to the available biomarkers, ST-segment elevation should raise clinical suspicion of ongoing ischemia post-operatively.

The polyester net heart positioning device is an elastic ventricular restraint device utilized for visualization during cardiac operations. The device assists in positioning the heart at optimum levels and allows access to the posterior side of the heart for grafting. We hypothesize that the placement of this device *via* the transverse sinus could have caused external compression of the ALCx stent that led to its subsequent occlusion, although this may have been too far cephalad. Alternative mechanisms include: distortion or compression of the retroaortic anomalous coronary from the aortic valve prosthesis (Figure 5), cessation of antiplatelet agents and reversal of heparin with protamine, or placement of the aortic cross clamp (also likely to be too far cephalad).

High clinical suspicion is indicated when dealing with anomalous coronary arteries. They are at increased risk during aortotomy for aortic valve replacement. Given the intraoperative external compression/manipulation, there may be a greater likelihood of occlusion due to unique structural abnormalities.

## What have we learned?

Congenital coronary artery abnormalities are present in 1% of the population. Patients with anomalous coronary arteries are at a greater risk of injury of their arteries during cardiac surgery, thereby increasing potential risk of ischemia and arrhythmias^2^. After performing cardiac surgery in patients with anomalous circumflex arteries, it is essential to monitor for new EKG changes that may be indicative of ischemia and necessitate urgent re-intervention.
